# Effects of UV Stress in Promoting Antioxidant Activities in Fungal Species *Тrametes versicolor* (L.) Lloyd and *Flammulina velutipes* (Curtis) Singer

**DOI:** 10.3390/antiox12020302

**Published:** 2023-01-28

**Authors:** Nenad Krsmanović, Milena Rašeta, Jovana Mišković, Kristina Bekvalac, Mirjana Bogavac, Maja Karaman, Omoanghe S. Isikhuemhen

**Affiliations:** 1 Department of Biology and Ecology, Faculty of Sciences, University of Novi Sad, Trg D. Obradovića 2, 21000 Novi Sad, Serbia; 2 Department of Chemistry, Biochemistry and Environmental Protection, Faculty of Sciences, University of Novi Sad, Trg D. Obradovića 3, 21000 Novi Sad, Serbia; 3 Faculty of Medicine, University of Novi Sad, Hajduk Veljkova 3, 21000 Novi Sad, Serbia; 4 Mushroom Biology & Fungal Biotechnology Laboratory, NRED, CAES, North Carolina A&T State University, Greensboro, NC 27407, USA

**Keywords:** UV exposure, phenolic compounds, AO activity, *Trametes versicolor*, *Flammulina velutipes*

## Abstract

*Trametes versicolor* and *Flammulina velutipes*, after submerged cultivation, with intermittent exposure to ultraviolet light (UV), were studied for Total Phenolic content (TP) and Total Proteins (TPR) contents and antioxidant properties against free radicals. The TP and TPR were determined by a spectrophotometric method and Lowry’s assay, respectively. Liquid chromatography with mass spectrometry (LC–MS/MS) was used to quantify polyphenols. Different in vitro assays determined the antioxidant activities of the extracts. Mycelia extract from *F. velutipes* after 14 days and filtrate from *T. versicolor* after 21 days of incubation gave the highest TP 59.60 ± 0.14 and 50.03 ± 0.66 mg GAE/g d.w., respectively. Mycelia extract from *T. versicolor* after 28 days of incubation had the highest TPR (183.53 ± 2.84 mg BSAE/g d.w.). The LC-MS/MS analysis indicated that *p*-hydroxybenzoic and protocatechuic acids are the most abundant. *Trametes versicolor* filtrate after 14 days and *F. velutipes* filtrate after 21 days (71.29 ± 0.54% and 73.5 ± 1.81, respectively) had the highest scavenging activity in SOA. Correlation analysis indicated that all extracts’ antioxidant (AO) potential strongly correlated with TP (R^2^ = 0.83–1.0). The data confirmed that stress factors such as UV exposure could stimulate the production of secondary metabolites and natural AOs, especially phenolic acids in test fungi.

## 1. Introduction

Ultraviolet (UV) radiation from the sun greatly impacts the environment, affecting nearly all living organisms. Biological reactions of organisms against the adverse effects of UV have evolved and sometimes show phenotypic effects, such as the production of pigments like melanin in fungi and animals [1,2]. Melanin is an example of the physiological value of both inducible and constitutive defense against UV radiation [3,4]. Melanin’s primary (evolutionary) function in microorganisms is to reduce the detrimental effects of UV radiation, e.g., DNA damage [3]. Although fungal melanin is not essential for normal growth, it enhances fungi’s survival and competitive abilities in stressful environments [5,6]. In indoor environments, commercially available low-pressure mercury-vapor lamps, usually used for sterilization, emit about 86% of their radiation in the mid-UV range (specifically 254 nm), with 265 nm being the peak of germicidal effectiveness. Damage in a microorganism’s DNA/RNA inhibits reproduction, even though the organism may not be killed [7]. The effects of UV radiation on fungal cells depend on the duration of UV exposure, wavelength, and irradiance of the incident photons that strike the cells [7]. At the cellular level, damage can be induced by direct absorption or indirectly, whereby UV is absorbed by intermediate compounds, which leads to the production of reactive oxygen species (ROS) that can cause damage to cellular components [8]. ROS, including superoxide anion radical (O_2_^•-^), hydroxyl radical (OH^•^), and hydrogen peroxide (H_2_O_2_), cause oxidative stress and have a destructive effect on various cell components [9,10]. Aerobic organisms have different mechanisms for producing many secondary metabolites in the mycelium and fruiting bodies in response to ROS production. Furthermore, their investigated biological effect varies depending on the chemical nature of metabolites and on the genetics of fungal species/strains [11]. 

The fruiting bodies, mycelia, and extracellular media from submerged cultivation can provide high quantities of bioactive compounds (primary and secondary metabolites) needed for oxidative stress defense mechanisms, which are of great relevance in basic and applied scientific research. In addition, the wide range of secondary metabolites produced by fungi makes them “mycofactories” that are extremely important in biotechnology and many other industrial processes [12].

Medicinal mushrooms *Trametes versicolor* (L.) Lloyd 1921 (*Polyporaceae*) and *Flammulina velutipes (Curtis)* Singer 1951 (*Physalacriaceae*) are saprotrophic species widely distributed across the world and readily found on dead hardwood logs and stumps. These species attract considerable attention due to their diverse biological activities and their broad spectrum of bioactive compounds [13,14,15,16,17,18,19,20,21,22]. 

*Trametes versicolor* contains bioactive proteins and polysaccharides (PSH), such as PSK and polysaccharopeptide PSP. Recently, 38 phenolic compounds (including flavonoids, hydroxybenzoic acids, hydroxycinnamic acids, and coumarins) have been detected in *T. versicolor* fruiting body [13]. Bioactive compounds differ depending on the species and their originshence their AO compound contents can be diverse: phenolics, polysaccharides, tocopherols, carotenoids, glycosides, ergothioneine, and ascorbic acid [10]. In comparison to extracts, mycelial biomass contains not only protein-bound polysaccharides (PSK; PSP) but also active enzymes responsible for (1) preventing oxidative stress, e.g., laccase, superoxide dismutase; (2) inhibiting cell growth, e.g., proteases, glucoamylases; and (3) promoting detoxification; e.g., peroxidases, cytochrome P450, and glucose-2-oxidase [23]. 

*Flammulina velutipes* is one of the most popular edible fungi in Asia. It contains high content of bioactive compounds such as polysaccharides, polysaccharide-protein complexes, sterols, and lectins. These compounds have various medicinal and pharmaceutical properties, including immunomodulating via induction of cytokines, antitumor, AO, thrombolytic, fibrinolytic, antibacterial, antifungal, and antiviral [15,17,18]. *Flammulina velutipes* and *T. versicolor* also contain polyphenols with many other bioactivities, such as AO and antimicrobial activity [10,13,14,16,20]. 

To our knowledge, no studies show the effects of UV exposure on *T*. *versicolor* and *F*. *velutipes.* Therefore, this research focused on changes in the production of primary and secondary metabolites after UV light exposure on submerged mycelial biomass and filtrate of these two fungal species.

## 2. Materials and Methods

### 2.1. Fungal Material 

*T. versicolor* and *F. velutipes* were collected in October 2020 from the Fruška Gora National Park (Republic of Serbia). The identification of the mushrooms was based on morphological and microscopic characteristics [13]. Mycelia used in this research were isolated from the fruiting bodies of both species and deposited in fungal culture collection FUNGICULT of the ProFungi laboratory at the Department of Biology and Ecology, Faculty of Sciences, University of Novi Sad, and referenced under the following numbers: 0071 and 0035, respectively, for *T. versicolor* and *F. velutipes*. 

### 2.2. Submerged Cultivation and Preparation of Extracts

The mycelia of both species were cultivated on Malt agar (Torlak, Serbia) at 25 °C for 14 days in a thermal incubator in the dark. The liquid fermentation medium used contained per liter 0.5 g peptone, 3.7 g glucose, 0.17 g maltose, 0.17 g fructose, 0.17 g xylose, 0.5 g yeast extract, 0.1 g K_2_HPO_4_, 0.05 g MgSO_4_ x 7 H_2_O and 0.005 g vitamin B_1_. Erlenmeyer flasks (EF) (500 mL wide throat glass flasks) containing 100 mL of sterile fermentation medium were inoculated with five plaques (1 cm^2^ in size) of *T. versicolor* and *F. velutipes* mycelia punched out of fully colonized agar plates. The incubation was done on a rotary shaker (IKA KS 4000i control, Werke GmbH and Co.KG, Staufen, Germany) at 120 rpm, 26 °C and in the dark for 7, 14, 21, and 28 days. 

Mycelia were grown on solid medium in glass Petri plates (ø140 mm), and liquid medium in Erlenmeyer flasks. Opened Petri plates and EF with removed plugs were exposed to UV light λ = 254 nm (Iskra IBK 1V2 laminar, Slovenia) for 2 h daily. The mycelial growth in Petri plates was measured daily using a ruler with graduations in millimeters (30 cm in length). At each sampling time, three replicates of both UV exposed and non- UV exposed mycelium (control) were removed from the incubator, and the contents were filtered (diaphragm vacuum pump GM-0.5, HINOTEK Group Limited, China). The recovered mycelia biomass and filtrates were stored in the refrigerator at 4 °C for 24 h, followed by lyophilization (filtrates = 72 h; mycelia = 48 h) at −80 °C (Bio alpha, Martin Christ GmbH, Switzerland). Lyophilized samples were ground to a fine powder (IKA A11 basic, Germany) and kept in amber bottles at room temperature until further use. For lyophilized mycelial biomass, ethanol extracts (EtOH) were prepared by mixing 2.5 g of fungal material with 50 mL of absolute ethanol (Zorka Pharma, Šabac, Serbia) and stirred at 120 rpm and room temperature for 72 h. Afterward, filtered extracts (diaphragm vacuum pump GM-0.5, HINOTEK Group Limited, Ningbo, China) were evaporated (Büchi R-210, Switzerland) until the dry weight (d.w.) was reached and redissolved in 80% EtOH to achieve a final concentration of 100 and 200 mg/mL. For lyophilized culture filtrate, ethanol extracts (EtOH) were prepared by adding 50 mL of absolute ethanol (Zorka Pharma, Šabac, Serbia) and stirred at 120 rpm and room temperature for 72 h. Afterward, filtrated extracts (diaphragm vacuum pump GM-0.5, HINOTEK Group Limited, Ningbo, China) were evaporated (Büchi R-210, Switzerland) until the dry weight (d.w.) was reached and redissolved in 80% EtOH to achieve a final concentration of 100 and 200 mg/mL.

### 2.3. LC-MS/MS Quantification of Selected Phenolic Compounds

The quantification of the selected phenolics in EtOH extracts of the studied species was carried out using the LC–MS/MS method [24]. Extracts were diluted with mobile phase solvents A (0.05% aqueous formic acid) and B (methanol), premixed in 1:1 ratio, to obtain a final concentration 2 mg/mL. Fifteen working standards, ranging from 1.53 ng/mL to 25,0103 ng/mL, were prepared by serial 1:1 dilutions of standard mixture with 50% methanol. Samples and standards were analyzed using Agilent Technologies high-performance liquid chromatograph (1200 Series) coupled with Agilent Technologies 6410A Triple Quad tandem mass spectrometer with an electrospray ion source and controlled by Agilent Technologies MassHunter Workstation software—Data Acquisition (ver. B.03.01). Five microlitres were injected into the system, and compounds were separated on Zorbax Eclipse XDB-C18 (50 mm 4.6 mm, 1.8 lm) rapid resolution column held at 50 C. The mobile phase (solvent A—0.05% aqueous formic acid and B—methanol) was delivered at a flow rate of 0.5 mL/min in a gradient mode (0 min 30% B, 12 min 70% B, 18 min 100% B, 24 min 100% B, re-equilibration time 6 min). Eluted components were detected by MS, using the ion source parameters as follows: nebulization gas (N2) pressure 30 psi, drying gas (N2) flow 9 L/min and temperature 350 °C, capillary voltage 4 kV, negative polarity. Data were acquired in dynamic MRM mode, using the optimized compound-specific parameters (retention time, precursor ion, product ion, fragmentor voltage, collision voltage) given in supplementary data S1. For all the compounds, peak areas were determined using Agilent MassHunter Workstation Software—Qualitative Analysis (ver. B.03.01). Calibration curves were plotted, and samples’ concentrations were calculated using the OriginLabs Origin Pro (ver. 8.0) software.

### 2.4. Determination of Total Phenolic (TP) and Total Protein (TPR) Contents

The total phenolic content (TP) was determined by the slightly modified method [25], which is based on spectrophotometric detection of phenols forming a colored complex with a Folin-Ciolcateu (FC) reagent. Lowry’s assay [26] determined the total protein content (TPR) in tested fungal extracts. The Lowry assay is based on the reaction of Cu^+^, produced by the oxidation of peptide bonds, with FC reagent. 10 µL of each extract or bovine serum albumin (BSA) standard solution (except in a blank probe where only the solvent was used, 80% EtOH), was added to 50 µL of distilled water (dH_2_O) and mixed with 100 µL of solution C (mixture of 2% sodium carbonate, 0.02% potassium sodium tartrate dissolved in 0.1 mol/L sodium hydroxide with hydrosoluble 0.5% copper sulfate) and 10 µL of solution E after 10 min. The absorbance was read at λ = 740 nm (Multiscan, Thermo Scientific, Waltham, MA, USA) after 30 min incubation time at room temperature. TPR was determined by comparison with the standard calibration curve of BSA, and results were expressed as milligrams of BSA equivalents per g dry weight of the extract (mg BSAE/g d.w.).

### 2.5. DPPH Radical Scavenging Assay

The radicals scavenging capability of the fungal extracts against DPPH radicals was determined using the slightly modified method described by Espin et al. [27]. The scavenging activity of extracts against DPPH radicals was defined as the percentage (%) of the initial DPPH population inhabited. Briefly, the reaction mixture contained 60 µL of 90 M DPPH reagent, 180 µL of methanol (MeOH), and 10 µL of the relevant EtOH extracts. The mixture was kept in the dark at room temperature for 30 min (modified from the original 60 min.), and the absorbance was measured at λ = 515 nm (Multiscan, Thermo Scientific, Waltham, MA, USA).

### 2.6. Hydroxyl Radical (OH^•^) Scavenging Assay 

The scavenging activity of OH^•^ was determined by the modified method of Halliwell et al. [28]. Formed reactive OH^•^ in the presence of 2-deoxy-D-ribose and oxygen react giving malondialdehyde (MDA), which is then determined using TBA (thiobarbituric acid) asay based on the spectrophotometric determination of pink colored complex. In a microtiter plate, 100 μL of 0.015% hydrogen peroxide, 100 μL of 10.0 mmol/L FeSO_4_ and 100 μL of 0.05 mol/L 2-deoxyribose-D-ribose were mixed with 10 μL of EtOH fungal extract and 2.7 mL of phosphate buffer (pH 7.4). After an incubation period of 60 min at 37 °C, the addition of 200 μL of 0.1 M ethylenediaminetetraacetic acid (EDTA) and 2 mL TBA reagent, heating on 100 °C and cooling, absorbance was spectrophotometrically measured at λ = 532 nm (Multiscan, Thermo Scientific, Waltham, MA, USA).

### 2.7. Superoxide Anion Radical Scavenging Assay (SOA)

This assay was determined according to the procedure of Nishikimi et al. [29]. Superoxide anion radical (O_2_^•-^), resulting from the univalent reduction of O_2_, is considered the first step leading to oxidative stress. Two systems are used for producing O_2_^•-^; the xanthine/xanthine oxidase (XOD) system and phenazine methosulphate (PMS) system in the presence of nicotinamide adenine dinucleotide (NADH). The reaction of the formation of O_2_^•-^ is based on the catalysis of XOD In this case, nitroblue tetrazolium (NBT), a probe/target for measuring the O_2_^•-^ scavenging capacities of samples, is used. The reaction mixture contained 200 μL of 144 µmol/L NBT, 10 μL of the extract, 100 μL of 677 µmol/L NADH, 100 μL of 60 µmol/L PMS and 1.1 mL of phosphate buffer (pH 8.3). The mixture was kept at room temperature for 5 min, and the absorbance was measured at λ = 560 nm (Multiscan, Thermo Scientific, Waltham, MA, USA).

### 2.8. Ferrous Ion Chelating Assay (FRAP)

The ferric-reducing AO power (FRAP) assay, according to Benzie and Strain [30] was used to evaluate the reducing power of the extracts. This procedure involves the reduction of Fe^III^ –TPTZ (Iron (II)-2,4,5-trypyridyl-*s*-triazine) at low pH values, to a blue-colored Fe^II^ –TPTZ, which increases absorbance at 593 nm. Briefly, 10 μL of extract solution or ascorbic acid (AA) standard solution at different concentrations was mixed with 225 μL FRAP reagent and 22.5 μL dH_2_O. The fresh FRAP reagent consists of 10 mmol/L TPTZ solution in 40 mmol/L HCl, 0.02 mmol/L FeCl_3_ × 6H_2_O, and acetate buffer (pH 3.6) in a ratio 10:1:1. This mixture was incubated in a microplate at room temperature for 6 min, and then the absorbance was measured spectrophotometrically at λ = 593 nm (Multiscan, Thermo Scientific, Waltham, MA, USA). This assay used ascorbic acid (AA) to calculate the standard curve. Reducing power was expressed as mg of ascorbic acid equivalents (AAE)/g d.w.

### 2.9. Statistical Analysis

All assays were performed in triplicates. The results are expressed as mean values ± standard deviation (SD). In scavenging in vitro AO assays, values represent the percent of inhibition (%) of analyzed extracts that cause neutralization/inhibition of free radical species. Correlation and regression analysis were also carried out using Microsoft Office Excel program for Windows, v.2007. The data that have a normal distribution were subjected to two-way analysis of variance (ANOVA) and multivariate analysis of variance (MANOVA). Tukey’s test was used to determine significant differences (*p* < 0.05) between the extracts. The statistical analysis was performed using the IBM SPSS Statistics software version 22.0 for Windows. Principal component analysis (PCA) provided an overview of the relationships between AO properties and AO components of irradiated and non-irradiated samples from *F. velutipes* and *T. versicolor*. PCA analysis was performed using XLStat 2022 software (Addinsoft, Inc., Brooklyn, NY), which provides the data’s internal structure and gives good data dispersion. 

## 3. Results аnd Discussion

### 3.1. Mycelial Growth 

**Figure 1:**
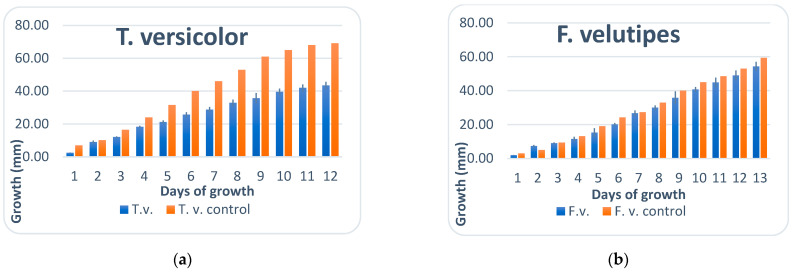
(**a**) T. versicolor: blue histograms represent growth of the UV exposed samples and the orange histograms control; (**b**) F. velutipes: blue histograms represent growth of the UV exposed samples and the orange histograms are the controls.

The growth of mycelium on solid media in Petri plates of each species (FCB 0071 and FCB 0035) was monitored for 2 weeks until mycelium reached the edges of the Petri plates (12 days and 13 days for *T. versicolor* and *F. velutipes,* respectively) and presented in Figure 1a,b as mean ± SD and control for both examined species.

After 12 days of cultivation, *T. versicolor* mycelia, exposed to direct UV irradiation, showed retarded growth, and diameter (45 mm) compared to the control (70 mm). Similarly, *F. velutipes* had 55 mm compared to 60 mm mycelia growth, indicating a negative impact of UV radiation on both strains. In addition, *T. versicolor* mycelia grown on agar plates appeared to be more sensitive to UV light exposure. 

### 3.2. Mycochemical Characterization

#### 3.2.1. LC–MS/MS Analysis

Only 10 compounds out of 45 standards used were detected and quantified using LC–MS/MS procedure (Table 1; Supplementary Material Table S1). The compounds were three hydroxybenzoic acids, two hydroxycinnamic acids, one cyclohexane carboxylic acid, two isoflavonoids, one biflavonoid, and one flavone (Table 1). The most abundant compound was *p*-hydroxybenzoic acid in the range of 8900–26,996.5 μg/g d.w and 45–2081 μg/g d.w for *F. velutipes* and *T. versicolor,* respectively. The highest amount of *p*-hydroxybenzoic acid was detected in the filtrate of *F. velutipes* extract, exposed to UV, after 21 days of incubation (26,996.50 μg/g d.w.) as well as quinic acid in the filtrate form both, treatment and control, of *T. versicolor* after seven days incubation (2081.0 and 984.5 μg/g d.w.). The highest amount of *p*-hydroxybenzoic acid found in the filtrate of *T. versicolor* exposed to UV, after 7 days of incubation, showed a concentration 13 times higher than reported for *T. versicolor* dry mushroom sample [16]. EtOH biomass extracts of *F. velutipes* contained most of the detected phenolic acids, whereas filtrate extracts showed higher phenolic content than mycelia biomass. Filtrate extracts of *T. versicolor,* after 28 days of UV exposure, showed much higher concentrations of biflavonoid amentoflavone (1934.5 μg/g d.w.) than the ones detected for EtOH biomass extracts not exposed to UV (7.79 μg/g d.w.) [13].

#### 3.2.2. Total Phenolic Content (TP)

Phenolic compounds are aromatic hydroxylated compounds with one or more aromatic rings and hydroxyl groups. They include phenolic acids, flavonoids, hydroxybenzoic acids, hydroxycinnamic acids, lignans, tannins, stilbenes, and oxidized polyphenols. Furthermore, many stimulate the synthesis of endogenous AO molecules in the cell [10,19]. It has been reported that phenolic compounds exhibit AO activity in biological systems, acting as free radical inhibitors, peroxide decomposers, metal inactivators or oxygen scavengers [10].

The results of TP content and TPR content are presented in Table 2. Based on the obtained results, it can be observed that the amount of TP in both species exposed to UV was rather different if filtrate and mycelia biomass are compared with the control. Extracts from *F. velutipes* contained higher TP content for mycelia biomass (54.24 ± 0.35 mg GAE/g d.w. and 59.60 ± 0.14 mg GAE/g d.w. for 7 and 14 days, respectively), while *T. versicolor* extracts contained a higher TP content in filtrate extracts (49.28 ± 0.83 and 50.03 ± 0.66 mg GAE/g d.w for 7 and 21 days respectively). This may indicate that UV exposure is *strain specific* and affects secondary metabolism differently. For example, *T. versicolor* is less sensitive to UV stress, resulting in higher extracellular production of TP in filtrate after ≈21 day (50.03 ± 0.66 mg GAE/mg d.w.). Additionally, *F. velutipes*, more sensitive to UV stress, reached secondary metabolism faster, resulting in the highest extracellular production of TP in filtrate after only two weeks (18.35 ± 0.33 GAE/mg d.w.).

Furthermore, *F. velitipes* UV light treated mycelia extracts after 7 days of incubation showed TP 54.24 ± 0.35 GAE/mg d.w., which was almost double than the control and had up to 3 times higher values than the ones reported for fruiting bodies of *F. velutipes* (16.69 ± 2.62 mg GAE/g extract) and many other culinary-medicinal mushrooms—*Auricularia auricula-judae* (6.19 ± 0.87 GAE/mg d.w.), *Hericium erinaceus* (10.20 ± 2.25 GAE/mg d.w.), *Lentinula edodes* (14.70 ± 3.01 GAE/mg d.w.) and several *Pleurotus* species (range from 9.26 ± 0.77–20.95 ± 2.39 GAE/mg d.w.) [18]. EtOH extracts from fruiting bodies of *T. versicolor* found in Turkey (9.58 mg GAE/g d.w.) [19] had almost five times fewer phenolics than *T. versicolor* extract after 21 days of exposure to UV light (50.03 ± 0.66 GAE/mg d.w.) in this study. In general, extracts that contain more phenolics express higher antiradical/antioxidant activity [31].

#### 3.2.3. Total Protein Content (TPR)

The TPR content in *T. versicolor* extracts from ranged from 64.80 ± 3.64–117.73 ± 2.83 mg BSAE/g d.w. for filtrate and 74.33 ± 0.36–183.53 ± 2.84 mg BSAE/g d.w. for mycelia biomass. The highest TPR content (after 28 days of treatment) was when mycelia biomass was the most abundant (Table 2). These results are in line with data in literature for *T. versicolor* from Serbia [13] and much higher than those found for fruiting bodies’ extracts (3.8 ± 0.8 mg BSAE/mL), as reported by Rašeta et al. [16].

The results obtained for EtOH mycelia biomass extracts of *T. versicolor* after 28 days of UV exposure (183.53 ± 2.84 mg BSAE/g d.w.) showed almost double values than the control (75.02 ± 0.86 mg BSAE/g d.w.), indicating a positive effect of UV irradiation on the production of mycelial biomass and its proteins. EtOH extracts from fruiting bodies of *T. versicolor* not exposed to UV were reported to have 98.10 ± 0.88 mg BSAE/g d.w. [20].

TPR content of *F. velutipes* ranged from 64.80 ± 3.64–133.16 ± 1.45 mg BSAE/g d.w. and from 8.56–98.65 ± 1.58 mg BSAE/g d.w. for filtrate and mycelia biomass extracts, respectively. For *F.velutipes*, UV negatively affected protein production since the maximum was in the first two weeks of cultivation, 133.16 ± 1.55 mg BSAE/g d.w. and 98.65 ± 1.58 mg BSAE/g d.w. for mycelia biomass and filtrate extracts, respectively, after which, it started to decrease. On the contrary, UV positively affected the production of proteins compared to the control.

### 3.3. In Vitro AO Activity

The most commonly used methods to measure AO activity in fungi involve chromogen compounds of radical nature that stimulate the reactive oxygen species (e.g., FRAP and DPPH methods). For the in vitro evaluation of the AO of EtOH extracts, the scavenging effect on 2,2-diphenyl-1-picrylhydrazyl radical (DPPH^•^) and ferric reducing AO power assay (FRAP assay) of extracts was determined. In addition, the capacity to neutralize superoxide anion (SOA, O_2_^•-^) and hydroxyl (OH^•^) was assessed for EtOH extracts. Results (Table 3) indicate that the best scavenging activity of OH^•^ and SOA assays was in the filtrate of *T. versicolor* and *F. velutipes* extracts, respectively, while reduction potential was noticed in *F. velutipes* extracts in general.

#### 3.3.1. DPPH Assay

All analyzed extracts exhibited low antiradical scavenging activity via DPPH assay (Table 3). Among *T. versicolor* EtOH extracts, the highest ability to capture DPPH radicals was shown by biomass extract after exposure to UV light for 21 days (RSC = 8.86 ± 0.13%) and after 28 days of cultivation (RSC = 8.56 ± 0.22%), while within filtrate extracts those incubated for 14 days showed results (RSC = 8.21 ± 0.12%) which is similar to results found by Rašeta et al. [16] and Mišković et al. [32]. For EtOH extracts of *F. velutipes*, the AO of filtrate increased from the 7th to 21st day when it had the highest activity (RSC = 7.08 ± 0.58%). Among biomass extracts, the analyzed scavenger activity had variations, but after 28 days, they proved to have the best ability to neutralize free radicals (RSC = 8.0 ± 0.05%). The differences in the DPPH scavenging activities exhibited by the various samples may indicate that the UV exposure, incubation period, and fungal species influenced the AO activity of the extracts. The extracts of mushroom species *T. versicolor* showed higher scavenging activity, and within this species, mycelia biomass extracts achieved RSC was (8.05–8.86%). There is no statistically significant difference if we compare values for mycelia biomass and filtrate of *T. versicolor* and *F. velutipes* with the control.

#### 3.3.2. SOA Assay

All analyzed EtOH extracts showed the ability to neutralize O_2_^•-^. The most potent was filtrate extract after 21 days for both tested species. Even though filtrate extracts showed much higher scavenging potential than mycelia biomass extracts, filtrate extracts from *T. versicolor* exposed to UV showed lower activity than those found earlier [16]. *T. versicolor* biomass and filtrate extracts, after 21 days of treatment, gave data that were almost double compared to the controls (Table 3). *F. velutipes* showed the same doubled values for filtrate extracts compared to the control but much earlier since it entered the secondary metabolism in the first two weeks.

#### 3.3.3. OH Assay

The results (Table 3) showed that the analyzed EtOH extracts of both species had high scavenging activities for the hydroxyl radical. EtOH extracts from *T. versicolor* showed the highest activity after 28 days of incubation for both mycelia biomass and filtrate extracts, RSC = 51.78 ± 0.61% and RSC = 36.36 ± 1.06%, repectively. EtOH extracts from *F. velutipes* mycelia biomass showed the best OH neutralizing activity after 28 days of incubation (RSC = 49.49 ± 2.62%), while filtrate extract displayed the highest activity after 21 days (RSC = 51.46 ± 0.02%). Both values are lower than the results obtained for EtOH extracts of *T. versicolor* [13]. Both species biomass and filtrate extracts showed better RSC activity than the control. Summarized, biomass extracts from T. *versicolor* exposed to UV were more effective than filtrate extracts. In contrast, both filtrate and biomass extracts from *F. velutipes* exposed to UV proved to be very effective, as presented in Table 3.

#### 3.3.4. FRAP Assay

The ferric-reducing ability of EtOH extract from tested species was evaluated and compared (Table 3). FRAP values for *T. versicolor* extracts were highest after 28 days for both filtrate (17.76 ± 0.01mg AAE/g d.w.) and mycelia biomass (26.79 ± 0.33 mg AAE/g d.w.). By comparing all of *F. velutipes* exposed to UV, both extracts, filtrate, and biomass, exhibited high activity after 14 days. Values obtained from UV exposed *F. velutipes* samples were higher than those published in 2014 by Yeh et al. (68.1 ± 1.07) for unexposed extracts [22]. Also, being stimulated by UV stress, earlier production of secondary metabolites for *F. velutipes* can be noticed after 14 days of exposure (85.36 ± 0.50), while control showed similar results after 28 days (83.10 ± 2.72). The extracts from *F. velutipes* could exhibit AO activity through free radical scavenging and reducing power, most likley due to phenolic compounds in the extracts. 

### 3.4. Correlation Analysis

A correlation analysis was performed to determine the relationship between TP and AO properties of analyzed extracts (Table 4). The highest correlation was detected between TP content and FRAP assay primarily for *F. velutipes* filtrates after 21 and 28 days of exposure to UV (R^2^ = 0.98 and R^2^ = 0.96, respectively) and all analyzed mycelia biomass EtOH extracts (R^2^ = 1). Also, high correlation was found for *T. versicolor* extracts for OH (R^2^ = 0.63–0.83) and DPPH assay (R^2^ = 0.73–0.99), primarily for mycelia biomass extracts. These results showed a statistically significant correlation between the scavenging activity, reducing power, and their content of phenolics. That emphasizes the importance of phenolic compounds and their influence on the total AO capacity [33].

In *F. velutipes*, a lower correlation between TP and analyzed AO assays was noticed. It could be explained by *F. velutipes* extracts showing lower TP content compared to *T. versicolor* extracts (Table 2). Most often extracts that contain more phenolics express higher AO and/or cytotoxic activities [33]. LC-MS/MS analysis (Table 1) revealed that within the strongest EtOH extracts beside *p*-hydroxybenzoic acid (26,996.5 μg/g d.w.), a high amount of protocatechuic acid was found (4501.5 μg/g d.w.). Results of OH assays for EtOH extracts are comparable with TP content since a high positive correlation was obtained (R^2^ = 0.99 and R^2^ = 0.91 for *T. versicolor* mycelia biomass and *F. velutipes* filtrate, respectively). Moreover, these results suggest the potential use of this mushroom for the prevention of oxidative stress since OH radical is physiologically active and toxic [27]. DPPH assay showed a statistically significant correlation with TP content, which is in accordance with previous data from submerged methanol samples of *Coprinus* species [34].

### 3.5. Principal Component Analysis (PCA)

**Figure 2:**
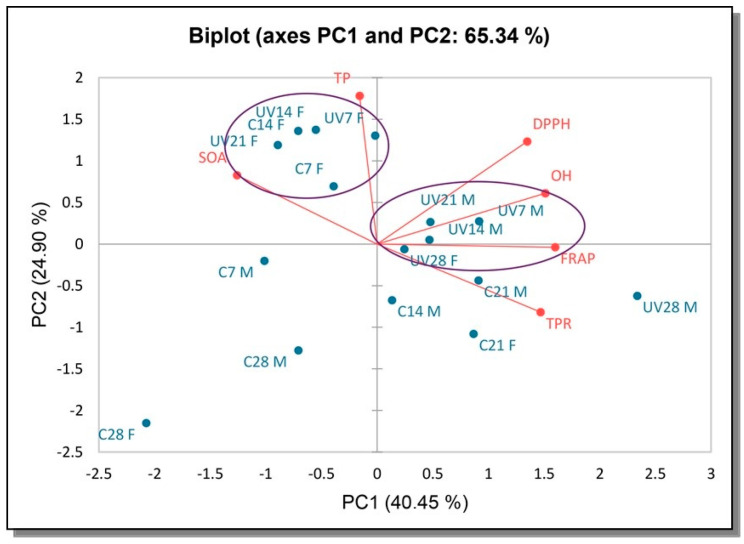
PCA of the AO properties and total contents of AO component of irradiated UV samples and control for *T. versicolor*. UV-samples exposed to UV irradiation, C-control, F-filtrate, M-mycelia biomass, 7, 14, 21, 28—days of incubation.

**Figure 3:**
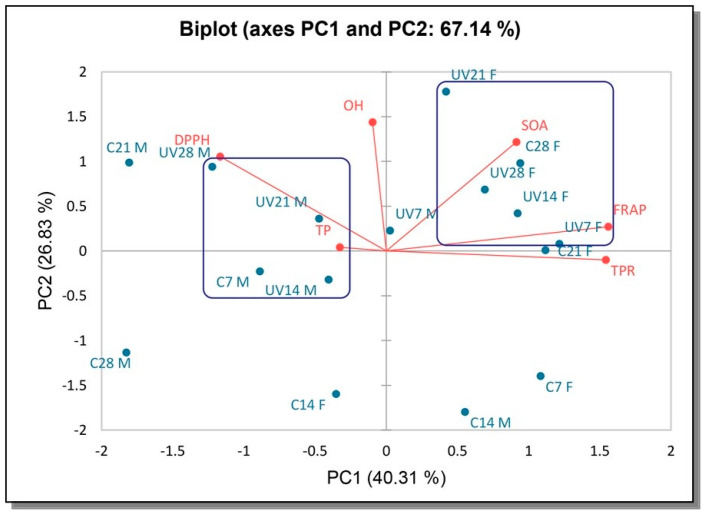
PCA of the AO properties and total contents of AO components of irradiated UV samples and control for *F. velutipes*. UV-samples exposed to UV irradiation, C-control, F-filtrate, M-mycelia biomass, 7, 14, 21, 28-days of incubation.

The multivariate treatment of the data was performed with the PCA analysis to gain an overview of the relationships among the AO properties and bioactive compounds formed by exposing the samples to UV irradiation during submerged cultivation (Figure 2 and Figure 3).

The multivariate treatment of the data obtained for the samples from *T. versicolor* permitted the reduction of the variables to two principal components, which together explained 65.34% of the total variability (Figure 2). The first axis (PC1) accounted for 40.45% and the second axis (PC2) for 24.90% of the total variability. Much stronger separation of the analyzed samples (40.45%) was obtained in the horizontal plane of the PC1 axis, with FRAP contributing the most. In addition, FRAP showed positive correlation with TPR while SOA showed a negative correlation with TPR.

For *T. versicolor* samples, a positive correlation between DPPH and OH was found. TP contributed to the greatest loading in the positive PC2 axis. We can notice several groups. The first group is in top right quadrant, with dominant samples for UV exposed biomass samples as opposed to the second group that is consisting of mostly UV exposed filtrate extracts (Figure 2). DPPH was responsible for the greatest loading in this quadrant positive for both axis, followed by OH.

The multivariate treatment of the data obtained for the samples from *F. velutipes* permitted the reduction of the variables to two principal components, which together explained 67.14% of the total variability (Figure 3). The first axis (PC1) accounted for 40.31% and the second axis (PC2) accounted for 26.83% of the total variability. The first group is composed of mycelia biomass extracts, mostly UV exposed after 7, 14 and 21 days. DPPH was responsible for the most significant loading in this quadrant, followed by OH. DPPH and OH seemed to correlate positively with TP, but negatively with TPR. The second group consists of mostly UV exposed filtrate extracts in the top right quadrant (Figure 3). Much stronger separation of the analyzed samples (40.31%) was obtained in the horizontal plane of the PC1 axis, with TP and TPR contributing the most. FRAP and SOA were responsible for the most significant loading in the quadrant with both PC1 and PC2 positive axis. They correlated positively with TPR, indicating their importance to the demonstrated activities. Results suggest that UV exposed samples have been mainly distributed via PC1 positive axis, unlike the Control samples.

In summary, irradiated and non-irradiated samples were well separated in score plots. Their AO properties and AO components were individually dispersed using PCA. FRAP, for both species, showed a positive correlation with TPR.

## 4. Conclusions

Most of the extracts exhibited AO, which is directly related to the characteristics of species growth as it can point to the moment of entry in the secondary metabolism. All tested EtOH extracts were able to neutralize O_2_^•-^ and had excellent scavenging activities for the hydroxyl radical. FRAP values for *T. versicolor* EtOH extracts were highest after 28 days of cultivation for both filtrate and biomass. OH, SOA, and FRAP activities for both tested species expressed best between 21 and 28 days of cultivation (Table 3). This observed quantitative and qualitative variability is expected considering that different origins of fungal growth (mycelia biomass, extracellular media) and effects of UV exposure during submerged cultivation can affect the production of different classes and concentrations of primary and secondary metabolites.

It was demonstrated that both species extracts exhibit AO activity, probably due to existing TP compounds found by LC–MS/MS phenolic analysis. The most abundant phenolic compound was *p*-hydroxybenzoic acid, protocatechuic acid, and quinic acid found in both species. This is the first report on the detection of biflavonoid amentoflavone in *F. velutipes* species. This is the first record of exposure of *T. versicolor* and *F. velutipes* mycelia to stress caused by UV irradiation. *T. versicolor* proved to be less sensitive to UV stress, which resulted in higher extracellular production of TP after 21 days, while *F. velutipes*, which we consider to be more sensitive, started production of secondary metabolites much earlier (within the first two weeks of cultivation). The differences in activities between two species and different types of extracts, together with variations of phenolics content, indicate the critical role of both genetic and environmental factors in metabolite production, and the possible synergistic effect of primary and secondary metabolites. Results show the potential of tested fungal species, *T. versicolor* and *F. velutipes*, to generate bioactive compounds (when exposed to UV light) that can be beneficial as food supplements to prevent damage caused by oxidation in the human body due to contamination or illness. Besides, mushroom AOs are of interest to the present generation as alternative healthcare compared to today’s expensive, high-tech disease treatment approaches.

## Figures and Tables

**Table 1:** Determined concentrations of selected phenolic compounds via LC–MS/MS method in examined EtOH extracts of *F. velutipes* and *T. versicolor* expressed as μg/g d.w.

		Class of Analyzed Compounds								
		Hydroxybenzoic Acid			Hydroxycinnamic Acid	Cyclohexanecarboxylic Acid	Isoflavonoid		Biflavonoid	Flavone
Analyzed Sample	Incubation Time (Days)	*p*-Hydroxybenzoic Acid	Protocatechuic Acid	Vanillic Acid	*p*-Coumaric Acid	Quinic Acid	Daidzein	Genistein	Amentoflavone	Apiin
*Flammulina velutipes*										
F	7	n.a.	n.a.	n.a.	n.a.	n.a.	n.a.	n.a.	n.a.	n.a.
	14	n.a.	n.a.	n.a.	n.a.	n.a.	n.a.	n.a.	n.a.	n.a.
	21	26,996.5	5405.0	18.0	8.0	63.5	642.0	440.5	0	0
	28	14,234.5	1759.5	70.0	6853.0	68.5	854.5	345.5	0	0
Fcont.	7	15,233.5	2261.0	17.0	75.5	63.5	1316.0	943.5	306	0
	14	n.a.	n.a.	n.a.	n.a.	n.a.	n.a.	n.a.	n.a.	n.a.
	21	n.a.	n.a.	n.a.	n.a.	n.a.	n.a.	n.a.	n.a.	n.a.
	28	19,580.5	4501.5	107.5	327.5	30	641	390	0	0
M	7	11,102.0	970	0	107	158.5	1702.5	3210.5	1115.5	114
	14	12,027.0	1475.5	21.5	106.5	81.5	1906.5	3152	0	266
	21	18,650,0	1555.0	56.5	52	72.5	1226	1966.5	0	85
	28	8900.0	3680.5	28	4756	43	1109.5	1304	0	89
M cont.	7	15,364.5	1475.5	21.5	102.5	204.5	2539	4824.0	0	1589
	14	n.a.	n.a.	n.a.	n.a.	n.a.	n.a.	n.a.	n.a.	n.a.
	21	n.a.	n.a.	n.a.	n.a.	n.a.	n.a.	n.a.	n.a.	n.a.
	28	n.a.	n.a.	n.a.	n.a.	n.a.	n.a.	n.a.	n.a.	n.a.
Range		8900–26,996.5	970–5405.0	17.0–107.5	8.0–6853.0	30–204.5	641–1906	345.5–4824.0	306–1115.5	85–1589
*Trametes versicolor*										
F	7	2081.0	19.5	0	36.5	341.5	189.5	148.5	0	0
	14	365	29	0	0	147.5	9	12	0	0
	21	275.5	33.5	0	0	127	13.5	0	782	96.5
	28	417.5	13	0	0	85	0	0	1934.5	0
F cont.	7	984.5	18.5	0	0	440.5	8	19.5	0	0
	14	275.5	36.5	0	0	84.5	5	2.5	404	0
	21	n.a.	n.a.	n.a.	n.a.	n.a.	n.a.	n.a.	n.a.	n.a.
	28	45	0	0	0	53.5	0	0	0	0
M	7	172.5	5.5	0	21	82	0	0	0	56
	14	n.a.	n.a.	n.a.	n.a.	n.a.	n.a.	n.a.	n.a.	n.a.
	21	83	7	0	11	70	5	5	0	25
	28	1200	26	0	0	170.5	4	13.5	594.5	140.5
M cont.	7	n.a.	n.a.	n.a.	n.a.	n.a.	n.a.	n.a.	n.a.	n.a.
	14	166	43.5	0	0	103	0	12	0	134.5
	21	n.a.	n.a.	n.a.	n.a.	n.a.	n.a.	n.a.	n.a.	n.a.
	28	262	11.5	29	0	48	5	35	0	104
Range		45–2081	5.5–43.5	0–29	11–36.5	48–440.5	4–189.5	5–148.5	404–1934.5	25–140.5

EtOH—ethanolic extracts; F—filtrate; M—mycelia biomass; n.a.—not analyzed, cont.—control.

**Table 2:** Total phenolic (TP) and total protein content (TPR) of EtOH extracts of *T. versicolor* and *F. velutipes*.

Extracts		TP (mg GAE/mg d.w.)		TPR (mg BSAE/g d.w.)	
*T. versicolor*		UV exposed	Control	UV exposed	Control
F	7 days	49.28 ± 0.83 ^a,c^	43.74 ± 0.45 ^a,c^	102.90 ± 2.20 ^a,c^	103.93 ± 3.59 ^a,c^
	14 days	48.43 ± 0.13 ^a,c^	47.22 ± 0.49 ^a,c^	79.40 ± 0.40 ^a,c^	64.80 ± 3.64 ^a,c^
	21 days	50.03 ± 0.66 ^a,c^	19.22 ± 0.22 ^a,c^	77.50 ± 1.50 ^a,c^	115.29 ± 1.11 ^a,c^
	28 days	18.78 ± 0.37 ^a,c^	8.61 ± 0.14 ^a,c^	103.16 ± 1.55 ^a,c^	117.73 ± 2.83 ^a,c^
M	7 days	47.36 ± 0.61 ^a,c^	12.67 ± 0.44 ^a,c^	122.98 ± 2.07 ^a,c^	74.33 ± 0.36 ^a,c^
	14 days	18.03 ± 0.39 ^a,c^	15.56 ± 0.30 ^a,c^	100.82 ± 0.50 ^a,c^	106.37 ± 3.27 ^a,c^
	21 days	17.86 ± 0.40 ^a,c^	15.94 ± 0.15 ^a,c^	99.15 ± 2.60 ^a,c^	114.95 ± 3.27 ^a,c^
	28 days	18.84 ± 0.35 ^a,c^	11.19 ± 0.33 ^a,c^	183.53 ± 2.84 ^a,c^	75.02 ± 0.86 ^a,c^
*F. velutipes*		UV exposed	Control	UV exposed	Control
F	7 days	17.26 ± 0.26^b,c^	17.81 ± 0.03 ^b,c^	112.90 ± 1.20 ^b,c^	103.93 ± 3.59 ^b,c^
	14 days	18.35 ± 0.33 ^b,c^	14.86 ± 0.55 ^b,c^	133.16 ± 1.45 ^b,c^	117.73 ± 2.83 ^b,c^
	21 days	16.98 ± 0.15 ^b,c^	16.69 ± 0.39 ^b,c^	99.50 ± 1.40 ^b,c^	95.29 ± 1.11 ^b,c^
	28 days	16.91 ± 0.11 ^b,c^	19.03 ± 0.39 ^b,c^	77.40 ± 0.0 ^b,c^	64.80 ± 3.64 ^b,c^
M	7 days	54.24 ± 0.35 ^b,d^	24.95 ± 1.28 ^b,d^	98.65 ± 1.58 ^b,c^	15.60 ± 0.88 ^b,c^
	14 days	59.60 ± 0.14 ^b,d^	58.34 ± 1.70 ^b,d^	75.58 ± 0.36 ^b,c^	19.67 ± 1.39 ^b,c^
	21 days	25.19 ± 2.65 ^b,d^	34.06 ± 0.96 ^b,d^	42.07 ± 2.25 ^b,c^	25.34 ± 0.17 ^b,c^
	28 days	21.94 ± 0.23 ^b,d^	13.71 ± 0.14 ^b,d^	8.56 ± 0.45 ^b,c^	14.01 ± 0.56 ^b,c^

Each value is expressed as mean ± SD. Means with different letters (a–d) within two columns per test (TP and TPR) are significantly different (Tukey’s HSD test and LSD post hoc test at *p* < 0.05) The differences are related to different species (*T. versicolor/F. velutipes,* letters a,b) and mycelia biomass (M) and Filtrate (F) (letters c,d), while there was no statistically significant difference between UV exposed samples and control. BSAE- bovine serum albumin equivalent; d.w.—dry weight.

**Table 3:** AO activity of EtOH extracts of *T. versicolor* and *F. velutipes*.

Extracts		DPPH (%)		OH (%)		SOA (%)		FRAP (mg AAE/g d.w.)	
*T. versicolor*									
		UV exposed	Control	UV exposed	Control	UV exposed	Control	UV exposed	Control
F	7 days	8.20 ± 0.11 ^a,c,e^	7.48 ± 0.22 ^a,c,e^	36.34 ± 2.45 ^a,c,e^	23.93 ± 0.60 ^a,d,e^	72.53 ± 0.38 ^a,c,e^	73.07 ± 2.23 ^a,c,e^	15.80 ± 0.21 ^a,c,e^	16.87 ± 0.20 ^a,c,e^
	14 days	8.21 ± 0.12 ^a,c,e^	7.95 ± 0.06 ^a,c,e^	24.65 ± 0.46 ^a,c,e^	26.79 ± 1.92 ^a,d,e^	73.55 ± 1.81 ^a,c,e^	71.90 ± 1.55 ^a,c,e^	15.43 ± 0.28 ^a,c,e^	15.55 ± 0.30 ^a,c,e^
	21 days	7.94 ± 0.09 ^a,c,e^	7.99 ± 0.15 ^a,c,e^	15.68 ± 2.00 ^a,c,e^	24.69 ± 0.19 ^a,d,e^	73.34 ± 1.14 ^a,c,e^	34.35 ± 0.52 ^a,c,e^	15.44 ± 0.42 ^a,c,e^	18.63 ± 1.30 ^a,c,e^
	28 days	7.88 ± 0.31 ^a,c,e^	3.96 ± 0.73 ^a,c,e^	36.36 ± 1.06 ^a,c,e^	N.A.	66.00 ± 0.48 ^a,c,e^	70.58 ± 0.10 ^a,c,e^	17.76 ± 0.01 ^a,c,e^	5.05 ± 0.27 ^a,c,e^
M	7 days	8.22 ± 0.41 ^a,c,e^	6.97 ± 0.14 ^a,c,e^	30.33 ± 1.15 ^a,c,e^	29.16 ± 1.91 ^a,c,e^	44.83 ± 1.24 ^a,c,e^	72.74 ± 0.10 ^a,c,e^	19.88 ± 0.34 ^a,c,e^	9.95 ± 0.21 ^a,c,e^
	14 days	8.36 ± 0.08 ^a,c,e^	8.70 ± 0.08 ^a,c,e^	30.88 ± 3.84 ^a,c,e^	4.98 ± 0.31 ^a,c,e^	69.43 ± 0.57 ^a,c,e^	58.45 ± 1.43 ^a,c,e^	24.11 ± 0.59 ^a,c,e^	22.85 ± 0.50 ^a,c,e^
	21 days	8.86 ± 0.13 ^a,c,e^	8.05 ± 0.47 ^a,c,e^	32.22 ± 1.98 ^a,c,e^	39.19 ± 2.96 ^a,c,e^	70.92 ± 1.72 ^a,c,e^	55.82 ± 2.10 ^a,c,e^	22.90 ± 0.40 ^a,c,e^	21.25 ± 0.90 ^a,c,e^
	28 days	8.56 ± 0.22 ^a,c,e^	4.29 ± 0.07 ^a,c,e^	51.78 ± 0.61 ^a,c,e^	22.82 ± 1.67 ^a,c,e^	57.57 ± 0.95 ^a,c,e^	68.15 ± 0.86 ^a,c,e^	26.79 ± 0.33 ^a,c,e^	24.89 ± 0.25 ^a,c,e^
*F. velutipes*									
		UV exposed	Control	UV exposed	Control	UV exposed	Control	UV exposed	Control
F	7 days	3.13 ± 0.52 ^b,c,e^	1.17 ± 0.18 ^b,c,e^	23.15 ± 1.57 ^a,c,e^	16.61 ± 1.96 ^a,c,e^	68.99 ± 0.70 ^b,c,e^	29.44 ± 0.30 ^b,c,e^	74.62 ± 0.09 ^b,c,e^	78.25 ± 1.10 ^b,c,e^
	14 days	4.53 ± 0.29 ^b,c,e^	3.10 ± 0.33 ^b,c,e^	24.55 ± 1.70 ^a,c,e^	8.04 ± 1.48 ^a,c,e^	68.06 ± 0.01 ^b,c,e^	25.46 ± 0.33 ^b,c,e^	85.36 ± 0.50 ^b,c,e^	23.72 ± 0.61 ^b,c,e^
	21 days	7.08 ± 0.58 ^b,c,e^	4.04 ± 0.05 ^b,c,e^	51.46 ± 0.02 ^a,c,e^	15.67 ± 2.11 ^a,c,e^	71.29 ± 0.54 ^b,c,e^	71.12 ± 0.31 ^b,c,e^	74.66 ± 0.23 ^b,c,e^	64.71 ± 1.74 ^b,c,e^
	28 days	5.25 ± 0.05 ^b,c,e^	7.26 ± 0.27 ^b,c,e^	30.58 ± 4.84 ^a,c,e^	25.40 ± 1.64 ^a,c,e^	71.16 ± 0.93 ^b,c,e^	71.45 ± 0.31 ^b,c,e^	56.08 ± 0.53 ^b,c,e^	83.10 ± 2.72 ^b,c,e^
M	7 days	6.23 ± 0.19 ^b,c,e^	7.00 ± 0.12 ^b,c,e^	26.10 ± 2.96 ^a,c,e^	N.D.	48.04 ± 1.70 ^b,c,f^	59.74 ± 2.79 ^b,c,f^	55.87 ± 0.36 ^b,c,f^	32.12 ± 1.65 ^b,c,f^
	14 days	7.04 ± 0.12 ^b,c,e^	2.90 ± 0.02 ^b,c,e^	12.53 ± 1.52 ^a,c,e^	N.D.	34.69 ± 2.01 ^b,c,f^	23.96 ± 1.39 ^b,c,f^	61.39 ± 0.14 ^b,c,f^	60.09 ± 1.75 ^b,c,f^
	21 days	6.60 ± 0.62 ^b,c,e^	8.14 ± 0.10 ^b,c,e^	42.68 ± 2.17 ^a,c,e^	39.96 ± 2.14 ^a,c,e^	37.75 ± 0.93 ^b,c,f^	40.21 ± 2.24 ^b,c,f^	25.94 ± 2.73 ^b,c,f^	35.08 ± 0.99 ^b,c,f^
	28 days	8.00 ± 0.05 ^b,c,e^	7.68 ± 0.32 ^b,c,e^	49.49 ± 2.62 ^a,c,e^	N.D.	41.36 ± 0.31 ^b,c,f^	21.12 ± 0.71 ^b,c,f^	N.A.	N.A.

Each value is expressed as mean ± SD Means with different letters (a–f) within two columns per test (DPPH, OH, SOA and FRAP) are significantly different (Tukey’s HSD, ANOVA, LSD, Friedman). Significant differences between extracts were determined by Tukey’s HSD test and LSD post hoc test at *p* < 0.05. The differences are related to different species (*T. versicolor*/*F. velutipes*, letters a,b), UV exposed samples and control (letters c,d) and Filtrate (F) and Mycelia biomass (M) (letters e,f). d.w.—dry weight; RSC (%) expressed on concentration of 100 µg/mL; SOA—superoxide anion radical assay; AAE—ascorbic acid equivalent; N.A.—non active; N.D.—in the examined range of concentrations, no activity was detected.

**Table 4:** Correlation between TP and determined AO properties of analyzed extracts.

		Correlation Coefficient—R^2^							
Extracts		DPPH/TP		OH/TP		SOA/TP		FRAP/TP	
*T. versicolor*									
		UV exposed	Control	UV exposed	Control	UV exposed	Control	UV exposed	Control
F	7 days	0.01	0.58 *	0.01	0.03	0.01	0.53 *	0.13	0.22
	14 days	0.73 *	0.45	0.63 *	0.23	0.61 *	0.26	0.91 *	0.08
	21 days	0.97 *	0.35	0.08	0.85 *	0.33	0.86 *	0.87 *	0.03
	28 days	0.01	0.48	0.44	/	0.44	1.00	0.05	0.21
M	7 days	0.99 *	0.70 *	0.83 *	0.93 *	0.73 *	0.08	0.88 *	0.09
	14 days	0.97 *	0.42	0.01	0.64 *	0.85 *	0.85 *	0.40	0.89 *
	21 days	0.74 *	0.36	0.76 *	0.42	0.02	0.97	0.16	1.00 *
	28 days	0.21	0.65*	0.40	0.99 *	0.40	0.16	0.01	0.82 *
*F. velutipes*									
		UV exposed	Control	UV exposed	Control	UV exposed	Control	UV exposed	Control
F	7 days	0.36	0.73 *	0.36	0.07	0.36	0.01	0.01	0.56 *
	14 days	0.04	0.54 *	0.56 *	0.91 *	0.10	0.18	0.02	0.04
	21 days	0.81 *	0.30	0.98 *	0.27	0.68 *	1.00 *	0.98 *	0.45
	28 days	0.01	0.22	0.01	0.08	0.01	0.48	0.96 *	0.59 *
M	7 days	0.55 *	0.93 *	0.67 *	/	0.01	0.99 *	1.00 *	0.16
	14 days	0.97 *	0.83 *	0.01	/	0.65 *	0.03	1.00 *	0.03
	21 days	0.12	0.47	0.40	0.43	0.12	0.88 *	1.00 *	1.00 *
	28 days	0.07	0.98 *	0.84 *	/	0.64 *	0.64 *	/	/

R^2^ *—All values are statistically significant (*p* < 0.05); F-filtrate; M-mycelia biomass; TP-total phenol content.

## Data Availability

The data are contained within the article and supplementary materials.

## Supplementary Materials

The following supporting information can be downloaded at: https://www.mdpi.com/article/10.3390/antiox12020302/s1, Table S1: Optimised dynamic MRM parameters.
