# Supplementary material for: Effects of UV Stress in Promoting Antioxidant Activities in Fungal Species Тrametes versicolor (L.) Lloyd and Flammulina velutipes (Curtis) Singer
Source: Antioxidants (Basel). 2023 Jan 28;12(2):302. doi: 10.3390/antiox12020302 (PMC9952144; doi:10.3390/antiox12020302)
Supplement: Supplementary file 1 [file antioxidants-12-00302-s001.zip › antioxidants-2155750-supplementary.pdf]

Table S1. Optimised dynamic MRM parameters.

compound	Precursor *m/z*	Product *m/z*	*Vfragmentor* (V)	*Vcollision* (V)	*tR* (min)
p-hydroxybenyoic acid	137	93	80	10	2.18
cinamic acid	147	103	100	5	10
protocathequic acid	153	109	105	9	1.94
2,5-dyhydroxybenzoic acid	153	109	100	9	2.64
umbeliferon	161	133	120	19	4.64
p-coumaric acid	163	119	90	9	5
o-coumaric acid	163	119	100	5	6.67
vanilic acid	167	108	100	15	3.35
gallic acid	169	125	90	10	1.36
esculetin	177	133	105	15	2.99
caffeic acid	179	135	100	10	3.18
quinic acid	191	85	150	20	1.09
scopoletin	191	176	80	8	3.28
ferulic acid	193	134	90	11	3.59
siringic acid	197	182	90	7	3.58
3,4-dymethoxycinamic acid	207	103	110	7	5.03
sinapinic acid	223	193	100	17	3.52
daidzein	253	208	145	31	6.4
genistein	269	133	145	32	7.69
apigenin	269	117	130	25	8.65
baikalein	269	269	165	0	10.1
naringenin	271	151	130	16	7.2
luteolin	285	133	135	25	7.45
kaempherol	285	285	130	0	8
catechin	289	245	150	10	1.91
epicatechin	289	245	150	10	2.91
chryzoeriol	299	284	125	20	8.96
quercetin	301	151	130	15	9
izorhamnetin	315	300	160	21	12.45
myricetin	317	179	150	20	6.97
chlorogenic acid	353	191	100	10	2.32
matairezinol	357	122	130	24	6.7
secoizolaricirezinol	361	165	130	26	5.29
vitexin	431	311	200	22	3.53
apigenin-7-*O*-Glc	431	268	135	41	5.07
baikalin	445	269	140	22	6.12

luteolin-7-*O*-Glc	447	285	230	30	4.02
quercitrin	447	300	190	27	5.23
kaempherol-3-*O*-Glc	447	284	190	30	5.3
epigalocatechin-galate	457	169	165	16	2.47
quercetin-3-*O*-Gal	463	300	200	30	4.08
quercetin-3-*O*-Glc	463	300	210	30	4.23
amentoflavon	537	375	220	35	15
apiin	563	269	250	36	4.82
rutin	609	300	135	42	4.14
